# Caspase Activation and Aberrant Cell Growth in a p53^+/+^ Cell Line from a Li-Fraumeni Syndrome Family

**DOI:** 10.1155/2015/789201

**Published:** 2015-03-18

**Authors:** Zaki A. Sherif

**Affiliations:** Department of Biochemistry and Molecular Biology, Howard University College of Medicine, Howard University, Washington, DC 20059, USA

## Abstract

Wild-type p53 is well known to induce cell cycle arrest and apoptosis to block aberrant cell growth. However, p53's unique role in apoptosis and cell proliferation in Li-Fraumeni Syndrome (LFS) has not been well elucidated. The aim of this study is to characterize the activity of wild-type p53 protein in LFS family dominated by a germline negative mutant p53. As expected, etoposide-treated wild-type p53-containing cell lines, LFS 2852 and control Jurkat, showed a greater rate of caspase- and annexin V-induced apoptotic cell death compared to the p53-mutant LFS 2673 cell line although mitochondrial and nuclear assays could not detect apoptosis in these organelles. The most intriguing part of the observation was the abnormal proliferation rate of the wild-type p53-containing cell line, which grew twice as fast as 2673 and Jurkat cells. This is important because apoptosis inducers acting through the mitochondrial death pathway are emerging as promising drugs against tumors where the role of p53 is not only to target gene regulation but also to block cell proliferation. This study casts a long shadow on the possible dysregulation of p53 mediators that enable cell proliferation. The deregulation of proliferation pathways represents an important anticancer therapeutic strategy for patients with the LFS phenotype.

## 1. Introduction

The Li-Fraumeni Syndrome (LFS) is a good model to study carcinogenesis in humans. This includes both quantitative and mechanistic studies. The syndrome is primarily characterized by the genetic lesion of the p53 protein, whose deleterious effects are manifested by the early onset of a wide range of malignancies. We are studying a unique family whose individual members are mostly affected by a heterozygous germ-line p53 mutation, which leads to the expression of a dominant negative form of p53 that effectively makes all the patients' cells p53 deficient. However, affected individuals carrying the heterozygous p53 would appear normal except for the development of primary cancers in numerous organs in early adulthood and young age. p53 is a tumor suppressor protein that functions as a sentinel of the genome by regulating cell growth and proliferation via cell cycle arrest and apoptosis. Its regulatory functions are facilitated by a number of downstream molecules that play key roles in cell cycle arrest such as p21; cell repair such as GADD45; and apoptosis such as Bax. There is also a host of newly discovered mediators that in turn control a varying number of proteins involved in cell growth and death [1–3].

p53 directs apoptosis following the activation of genes involved in redox reactions; the creation of free radicals, and the oxidative debasement of mitochondrial components that will result in cell death as reported by Polyak and colleagues [4]. The tumor suppressor protein p53 is located in the nucleus and promotes apoptosis in response to death stimuli by activating target genes and by transcription-independent mechanisms. p53 can specifically translocate to mitochondria, where it physically interacts with and inactivates Bc1-2 proteins that promote survival. Cells that are resistant to p53-dependent apoptosis have shown that p53 mediates cell death predominantly via the intrinsic death pathway [5].

Apoptosis is triggered by a number of dominant oncoproteins such as c-Myc, which may play dual roles by promoting cell proliferation and apoptosis in disparate pathways that may be intimately linked [6]. Apoptosis is crucial for vital cell processes including normal embryonic development, cell signaling, proper immune response, and cell death when necessary. Therefore, it can appear at any stage of the cell cycle since the metabolic process of the cell is omnipresent [7]. Sometimes, apoptosis may be triggered by external induction of the cell's operation as shown in prostate cancer cells during treatment with thapsigargin, which enables the release of Ca^++^ from the inner stores of the endoplasmic reticulum and results in a related incursion of extracellular calcium in the cell. Cells are then arrested at G_0_ 24 hours following cessation of cell-cycle progression whose ultimate elevation of intracellular calcium leads to apoptosis with its signature morphological changes [7].

Morphologically, the features of mitosis are quite similar to apoptosis: cell contraction, chromatin condensation, DNA fragmentation, and membrane blebbing as well as degradation of mitochondria. Moreover, critical tumor suppressor genes such as p53 and RB may determine the fate of cells in both cell cycle and apoptotic pathways. Therefore, it is evident that the equilibrium between proliferation and apoptosis must be stringently regulated to ensure tissue homeostasis, a prerequisite for avoiding carcinogenesis and a posttreatment relapse [3].

Cell proliferation, on the other hand, is a characteristic feature of tumor cell lines. These cells lose cycle control mainly as the result of p53, Chek2, or RB mutations. The function of such tumor suppressor genes is to monitor aberrant cell growth as a result of DNA damage. When a critical tumor suppressor gene such as p53, which is regarded as the custodian of the genome, is itself mutated as is the case in 50% of all human cancers and in about 70% of LFS patients, a damaged DNA in a cell will divide and multiply as the aberrant cell grows in a control-free environment. It was therefore intriguing as to why the LFS wild-type p53 harboring cell line, 2852, which has a functional homozygous p53 that induces apoptosis, will also proliferate at an abnormal rate. We therefore proceeded to analyze apoptotic events in different subcellular components of the two LFS cell lines, 2852 (p53-wild-type homozygous) and 2673 (p53-mutant heterozygous), to understand the specific role of p53 in LFS and its relationship to carcinogenesis.

## 2. Materials and Methods

*Ethics Statement.* The Howard University IRB (Internal Review Board) approved the use of the cell lines described herein and determined the protocol to be exempt based on* 45 CFR 46.101(b)(4)* and has stated that it involves minimal risk. The Li-Fraumeni Syndrome (LFS) cell lines, which are unidentifiable, were generously provided by NCI's Frederick Repository through Dr. Alice Goldstein. This established cell lines were first characterized by Joseph Fraumeni, Jr., and so forth [8, 9].

### 2.1. Cell Lines

Early passage normal LFS human skin fibroblast (noncancerous) cell lines 2673 (heterozygous mutant-p53) and 2852 (homozygous wild-type-p53), were acquired from Dr. Alice Goldstein (NCI Repository, Frederick, MD). These cells and unrelated wild-type p53-containing primary foreskin fibroblasts were maintained in MEM (Earle's salts and L-glutamine) with 10% FBS and 25 mM HEPES buffer in accordance with a previously published protocol [10]. For apoptosis studies, the fibroblasts were seeded into six-well culture dishes in a density of 3 × 10 cells/well [6]. The dose used for each apoptosis inducing agent is also indicated in Section 3. All cells were kept at 37°C and 5% CO_2_ as indicated previously [10].

### 2.2. p53 Activity

As indicated previously (see [10]), both 2673 and 2852 cells were treated with 100 or 150 *μ*M of the topoisomerase inhibitor etoposide for varied times up to 48 h, and apoptotic conditions in the two LFS cell lines were identified by standard 0.5% DNA fragmentation electrophoresis and a Cell Death Detection ELISA Kit (Roche Diagnostics Corp.), as previously indicated [10]. Briefly, DNA fragmentation was analyzed by 0.5% gel-electrophoresis and by nucleosomal ELISA kit after exposure to 150 *μ*M of etoposide at different time intervals of 0, 24, and 48 hours and a representative experiment of three independent replicates was carried out [10].

### 2.3. Caspase Assay

Induction of apoptosis with 150 *μ*M etoposide was carried out for up to 72 hours per cell line. Staining kits from CaspGLOW in situ or AFC fluorometric assay from Biovision (Mountain View, CA) were employed for detecting activated caspases in the cytoplasm of living cells in three replicate plates containing either 2852 or 2673. The assays were employed with strong caspase inhibitors conjugated to FITC as the fluorescence in situ markers. Activated caspases in apoptotic cells were directly visualized by fluorescence microscopy using FITC-marked labels. In addition, caspase inhibitors lacking FITC labels were also incorporated in the kits as negative controls. For the detection of early stages of apoptosis, the annexin V reagent from Biovision was utilized. During apoptosis, phosphatidylserine (PS) is translocated from the cytoplasmic face of the plasma membrane to the cell surface. Annexin V has a strong, Ca^2+^-dependent affinity for PS and therefore is used as a probe for detecting apoptosis. The annexin V-FITC conjugate can be used for detection of apoptosis by fluorescence microscopy or by flow cytometry.

### 2.4. Mitochondrial Assay

Detection of change in mitochondrial transmembrane potential was measured by MitoCapture probe and fluorescence microscopy using the instructions by the commercial kit provider (Biovision, Mountain View, CA). Cells were scored for intact and apoptotic cells using MitoCapture probe. Apoptotic cells should show diffused green fluorescence, whereas the normal control cells show punctate red fluorescence [11]. Incidentally, this protocol even with repetition showed neither diffused green fluorescence nor punctate red fluorescence according to the manufacturer's instructions.

### 2.5. Nuclear Assay

DNA ladder detection kit and custom service from Biovision were used to assay for nuclear stability following apoptosis induction.

### 2.6. Cell Proliferation Assay

Cells (2000 cells/well) were split into 96-well plate in regular growth medium plus 10% FBS. For four consecutive days, cell densities were measured every 24 hours using the Quick Cell Proliferation Assay Kit (Biovision, Cat. number K301-2500) according to the instructions of the provider. Four wells for each cell type were measured on each time point. The data represent the average O.D. reading from 4 individual wells.

### 2.7. Statistical Parameters

For each experiment involving a caspase activity assay or annexin V apoptosis assay, the cells were subjected to their respective treatments at different time intervals of 0, 24, 48, and 72 hours as representative experiments of three independent replicates. The cell proliferation assay was similarly conducted but with four independent replicates for each cell line. All data were analyzed for statistical significance and were represented in graphs with standard errors and calculated probabilities.

## 3. Results

### 3.1. Etoposide-Induced DNA Fragmentation of Human Fibroblast Cancer Cell Lines 2852 and 2673

Initially, five different apoptosis inducers (actinomycin D, etoposide, camptothecin, cycloheximide, and dexamethasone) were used from 6 hours to 36 hours to induce apoptosis in both 2852 (wt-p53) and 2673 (mt-p53) LFS cells. After a long period of optimization procedures, etoposide at 150 *μ*M seemed to be the most effective dose across the different cell types. The presence of a functional p53 in the LFS cell lines, 2852 and 2673, was verified by DNA fragmentation analysis (see Figure 4A in [10]) and apoptotic assay (see Figure 4B in [10]). Etoposide is a topoisomerase inhibitor that acts as a chemotherapeutic drug by inducing single- and double-stranded DNA breaks in the presence of a functional p53 and thereby disrupting cells in the G1 phase of the cell cycle [12]. The results of the caspase activity experiment in this current study confirmed that cell lines 2852 and 2673 displayed distinct time-dependent susceptibilities to etoposide [10]. The p53 wild-type-harboring 2852 cells were highly responsive to apoptosis in the first 24 h following etoposide treatment and even showed a significantly greater sensitivity at 48 h (*P* < 0.0001). On the other hand, the mutant 2673 cell line showed an apoptotic response rate barely above the basal line. This result was expected and conclusively demonstrates the presence of an active p53 protein in cell line 2852 and its absence in 2673.

To clearly understand whether this weaker activity of p53 from LFS cells was characteristic of the unique genetic syndrome of LFS or a phenomenon shared by other human cancer cell lines, we utilized Jurkat cells (immortalized human T lymphocyte cells) to conduct more caspase activation assays. The results as measured by annexin V and caspGLOW assays (data not shown) showed induction of a strong apoptosis with 150 *μ*M etoposide treatment. It is also important to note here that the LFS cells in general grew extremely slowly in the presence of etoposide when compared to Jurkat cells.

### 3.2. Caspase Fluorometric Assay

**Figure 1:**
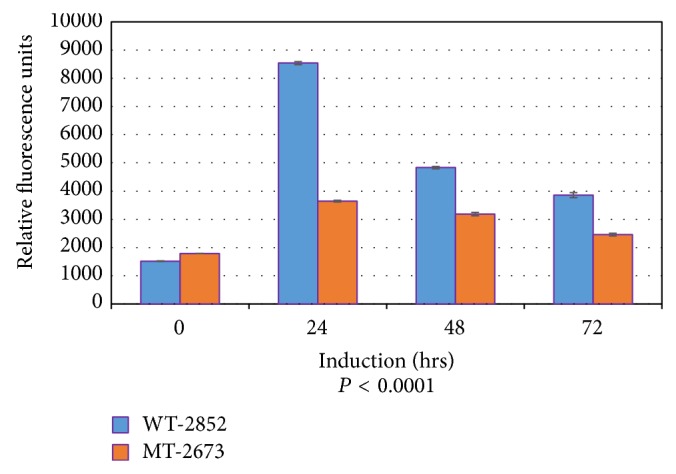
Caspase activity assay after 60 minutes of reaction. Apoptosis was induced by adding 150 *μ*M (final concentration) etoposide into culture medium for various hours as indicated. Medium was replaced with fresh etoposide every 24 hours. Cells were collected by trypsinization. Apoptosis was analyzed using Caspase-3 Fluorometric Assay kit (Cat. number K105-100) from Biovision (Mountain View, CA) according to the manufacturer's instructions. Results were analyzed by fluorometer. Data were shown as relative fluorescence units (shown on the *y*-axis). A representative experiment of three independent replicates is shown.

**Figure 2:**
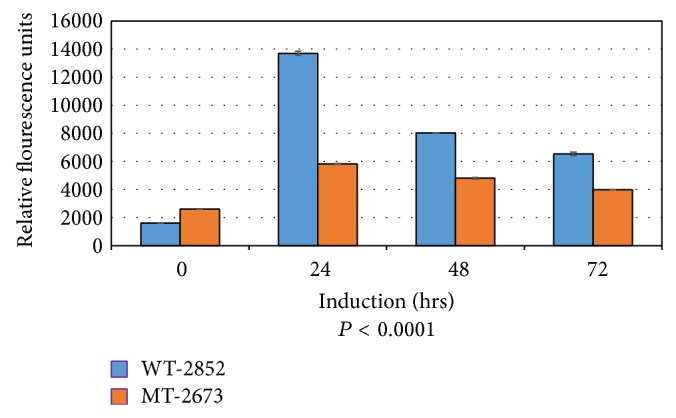
Caspase activity assay after 120 minutes of reaction. Apoptosis was induced by adding 150 *μ*M (final concentration) etoposide into culture medium for various hours as indicated. Medium was replaced with fresh etoposide every 24 hours. Cells were collected by trypsinization. Apoptosis was analyzed using Caspase-3 Fluorometric Assay kit (Cat. number K105-100) from Biovision (Mountain View, CA) according to the manufacturer's instructions. Results were analyzed by fluorometer. Data were shown as relative fluorescence units (shown on the *y*-axis). A representative experiment of three independent replicates is shown.

To determine whether caspase is activated during p53-dependent apoptosis in LFS cells, 2852 (wild-type p53) and 2673 (p53-mutant), 150 *μ*M etoposide was added to the culture medium, which was replaced with fresh etoposide every 24 hours. Cells were collected by trypsinization and apoptosis was analyzed using Caspase-3 Fluorometric Assay kit as described in Section 2. Although the results for both 2852 (wt-p53) and 2673 (mt-p53) cells showed initial caspase activity, which generally shows a transient pattern (consistent with assays using other systems such as Jurkat cells), the caspase activity as a measure of apoptosis induction, increased drastically (almost twofold) in 2852 cells after 24 h following treatment with etoposide whereas the caspase activity in 2673 cells was negligible (*P* < 0.0001) (Figure 1). Apoptosis was measured by caspase activation in relative fluorescence units. The cell lines from the two LFS siblings displayed varying time-dependent susceptibility levels to etoposide. This was shown in both the 60-minute (Figure 1) and 120-minute (Figure 2) reactions, respectively. The p53 wild-type 2852 cells were highly responsive to etoposide treatment with significantly higher apoptosis than in 2673 cells in both the initial 24 h and 48 h treatments. These results clearly demonstrate with respect to LFS cell lines that caspase is activated unambiguously in those cells actively undergoing p53-dependent apoptosis because of the presence of a wild-type homozygous p53; whereas there is little expression of p53 in a matched cell line that also harbors a p53 dominant negative mutation. The raw data of triplicate experiments for each cell line and for each reaction time are presented in Tables 1 and 2 of the Supplementary Material available online at http://dx.doi.org/10.1155/2015/789201.

### 3.3. Annexin V Apoptosis Assay

**Figure 3:**
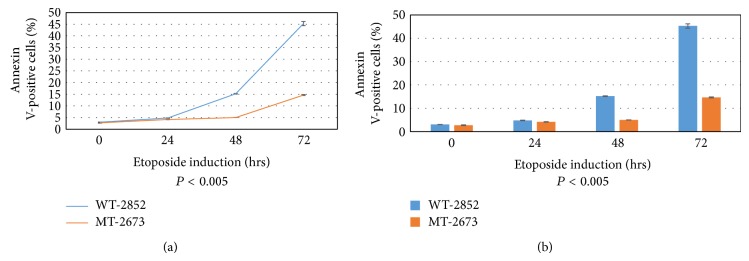
Annexin V apoptosis assay. Apoptosis was induced by adding 100 uM (final concentration) of etoposide into culture medium containing noncancerous LFS fibroblasts 2852 and 2673, which were incubated for various hours as indicated. Apoptosis was analyzed using Annexin V-FITC Apoptosis Assay Kit (BioVision, Cat. number K101-100) according to the instructions in the kit. Results as shown in line graph (a) and bar graph (b) were analyzed on FACScan with 10000 cells being counted from each sample. A representative experiment of three independent replicates is shown.

Apoptosis was induced by adding 150 *μ*M (final concentration) of etoposide into culture medium for various hours as indicated. During apoptosis, phosphatidylserine, an essential component of phospholipid membrane, is translocated from the cytoplasmic face of the plasma membrane to the cell surface. Annexin V is used as a probe for detecting apoptosis because it has a robust Ca^2+^-dependent affinity for phosphatidylserine. Apoptosis was analyzed using Annexin V-FITC Apoptosis Assay kit as detailed in Section 2. Results were analyzed on FACScan with 10,000 cells being counted in each sample. As shown in Figures 3(a) and 3(b), the etoposide-induced apoptosis using annexin V staining procedure sharply increased by a factor of three, 48 h following treatment, and by a factor of four, 72 h after etoposide treatment (*P* < 0.005). The data were graphed in two ways: line graph (Figure 3(a)) and bar graph (Figure 3(b)) for clarity. The activation of caspase is a good indicator of the intrinsic pathway of apoptosis, which might also activate the PARP enzyme triggered after double-strand DNA breaks [13].

The raw data of triplicate experiments for each cell line are presented in Table 3 of the Supplementary Material.

### 3.4. Mitochondrial and Nuclear Assays

It was necessary to determine apoptosis in the different organelles of living cells because inducers of apoptosis working through the mitochondria death pathway are now surfacing as potential drugs in several types of tumors [14]. The MitoCapture Apoptosis Detection kit that was utilized to assess apoptosis in the mitochondrion and the nucleus of living LFS cells produced negative results. The MitoCapture signal accumulates in the mitochondria of healthy cells fluorescing bright red, whereas, in apoptotic cells, it stays in the cytoplasm by fluorescing green. These distinct fluorescent signals can be readily identified by fluorescence microscopy or flow cytometry. However, there was no detection of apoptosis through repeated procedures in the mitochondria. The nuclear assay did not work either.

### 3.5. Cell Proliferation Assay

**Figure 4:**
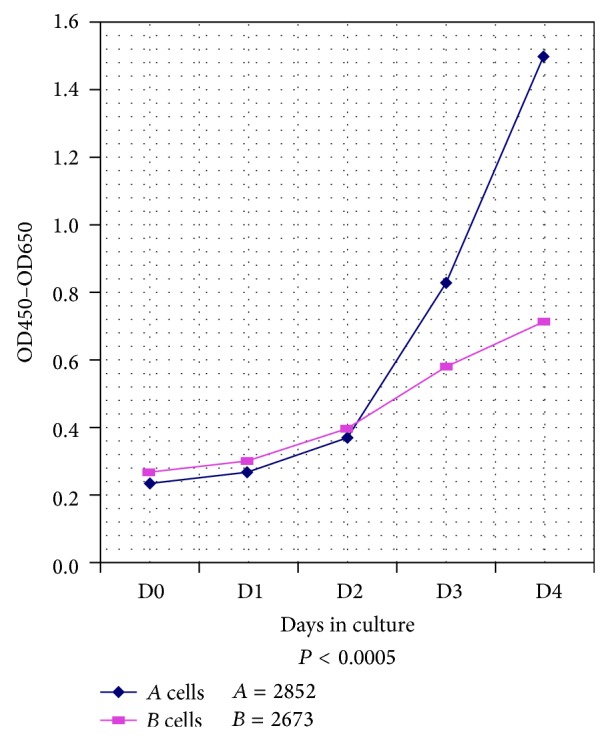
Cell proliferation assay. Comparison of cell proliferation between 2852 and 2673. Cell density was measured every 24 hours for 4 days using the Quick Cell Proliferation Assay kit (BioVision, Cat. number K301-2500). The data represent the average O.D. reading from 4 individual wells for each cell line.

As stated in Section 2, cell density as a measure of cell proliferation was measured every 24 hours for four straight days using the Quick Cell Proliferation Assay Kit (see Section 2). The data in Figure 4 represent the average O.D. reading from 4 individual wells for each cell line. It was unexpected that a wild-type-p53-containing cell line (i.e., 2852) would proliferate faster than the mutant-p53 harboring cell line (i.e., 2673). As Figure 4 clearly shows, at the end of the 4-day growth cycle, 2852 cells grew almost twice as fast as the 2673 cells (*P* < 0.0005). In an earlier cell cycle study involving serum starvation, the p53 protein was able to arrest cells at GI phase of the cell cycle with a concomitant induction of the cell growth inhibitor, p21 [10]. The raw data of quadruplicate experiments for each cell line are presented in Table 4 of the Supplementary Material.

## 4. Discussion

In this study, the two LFS cell lines, which were derived from normal skin fibroblasts of two siblings with different p53 status and cancer types, may have widely varied genetic background and genetic alterations in addition to the p53 genotype. Nevertheless, it has been demonstrated in this study that the wild-type p53-containing fibroblast cell line not only has the machinery for induction of apoptosis as would be expected of a normal p53 but also exhibited a surprisingly high proliferating characteristic. The data clearly show that apoptosis was triggered by caspase activity in a p53-dependent manner even though caspase was being degraded following activation, while the cells continued to undergo apoptosis beyond the 24 h induction period. Similarly, in an earlier published study involving the same cell lines, differential etoposide sensitivities were also observed by FACS analysis, which demonstrated that 2852 cells had started to accumulate in G1 cycle in the first 24 h of treatment in stark contrast to 2673 cells, which showed no significant sequel on cell cycle even after 48 h (see Figure 3 in [10]).

This research was initiated following frequent observations of faster cell growth rates for 2852 cells in tissue culture medium during the course of our cancer research studies. Although the cell line was developed from the normal noncancerous skin fibroblasts of a bilateral breast cancer patient, its growth characteristics were similar to those of transformed cells. The other noncancerous fibroblast cell line, 2673, developed from its sibling who had brain cancer, exhibited a less drastic growth pattern in close-proximity to that of a human non-LFS cancer cell line with an intact p53 protein and an NIH 3T3 mouse cell line [10]. This prompted the detailed study of apoptosis and cell proliferation in these cell lines to determine whether this was peculiar to Li-Fraumeni Syndrome cells. Review of the literature indicates that the wild-type containing LFS cell line, 2852, undergoes apoptosis in response to radiation [15]. However, other apoptosis markers have not been associated with this LFS family under study. Treatment of the cell lines with a known protein synthesis inhibitor, cycloheximide, or any of the other four DNA-damaging agents (see Section 3) did not produce apoptosis in these LFS cells. Etoposide seems to be the only agent between 100 *μ*M and 150 *μ*M final concentration that induced apoptosis in the cells. Although, DNA fragmentation was observed in 2852 as opposed to 2673 as expected [10], the morphological changes associated with apoptosis including degradation of mitochondrial components were not observed in these cells. These cells were quite unique in the fact that they did not respond to apoptosis induction readily even using the analytical tools of annexin V and caspase assays (two of the most widely employed and well-studied markers of apoptosis). The activation of caspase is crucial to apoptosis. It is, however, possible that these unique cells respond to apoptosis inducing agents possibly due to lesions present in the nonfunctional or unused pathway and using either one or the other classes of caspases: the initiators that include caspase-8, caspase-9, and caspase-10 or the effectors that predominantly work through caspase-3. Moreover, there are at least two divergent apoptotic signaling pathways: one, which is activated by the release of cytochrome c, which in turn activates caspase-9/Apaf1/cytochrome c apoptosis pathway; and the other invariably mediated by death receptors via caspases-8/10 [5]. Although direct cytochrome c release was not measured, the failure of degradation in the mitochondrial components of the cells suggests that the latter pathway was primarily used to affect apoptosis in the cells. This may be more likely in light of recent data demonstrating the frequent activation of caspase-8 and its substrate to implement apoptosis in response to specific stimuli triggered in a death receptor-independent manner [16, 17]. A recent cytogenetics study also revealed a rare type of chromosomal translocation involving chromosomes 11 and 15 in 2852 cell line [18]. Although the bilateral breast cancer patient (i.e., 2852) had a functional wild-type p53 protein as evidenced by the p53-dependent apoptosis, it is apparent that other genetic events possibly precipitated by the chromosomal rearrangements that have been observed are at play. These results also suggest the existence of a high level of cross-talk between these two distinct signaling pathways in order to coordinate events unique to the particular cell. This study is the first that is known to the author that details optimal conditions and agents for induction of apoptosis in LFS fibroblast cells that are resistant to apoptotic induction initiated by an array of chemotherapeutic drugs.

Similarly, we recently reported in an indirect way that p53 apoptotic activity is indeed decreased in these cells and that this correlated with a loss of Caveolin-1 (Cav-1) expression [10]. Caveolin is a putative tumor suppressor protein that is involved in lipid transport and signal transduction. Conversely, it was shown that p53 and Cav-1 levels increase in cells from non-LFS individuals in response to activators of p53 activity using serum starvation and etoposide-dependent apoptosis [10].

Cell cycle control and apoptosis, two cardinal events associated with p53 also work closely together. This has been shown by the restoration of p53 function, which led to the downmodulation of Bc1-2 levels and the manifestation of apoptosis [19]. On the other hand, high levels of Bax messenger RNA (mRNA) and protein are produced when p53 activates the Bax promoter [20, 21]. Annexin V and caspase are two well-studied markers for apoptosis studies. Therefore, it is evident that the stable existence of functional genes regulating cell cycle and apoptosis in a balanced manner is a prerequisite for controlling the rate of cell division and apoptosis that aligns with the needs of a normal cell. Consequently, loss or malfunction of any of these critical genes such as p53 whose activity may be influenced by the gain of function of the dominant negative (mutant) gene or protein that might lead to abrogation of control in the checkpoint domain and may result in an increase in cell proliferation.

## Supplementary Material

This supplementary material contains raw data for the apoptotic assays induced by caspase (60-minute reaction in Table 1, and 120-minute reaction in Table 2) and Annexin V (Table 3). Table 4 provides data for the cell proliferation assay (Table 4). Standard errors and calculated probabilities were derived from these data sets.

## References

[1] Vogelstein B., Lane D., Levine A. J. (2000). Surfing the p53 network. *Nature*.

[2] Zhang X., He Y., Lee K.-H. (2013). Rap2b, a novel p53 target, regulates p53-mediated pro-survival function. *Cell Cycle*.

[3] Daniele S., Taliani S., Da Pozzo E. (2014). Apoptosis therapy in cancer: the first single-molecule co-activating p53 and the translocator protein in Glioblastoma. *Scientific Reports*.

[4] Polyak K., Xia Y., Zweier J. L., Kinzler K. W., Vogelstein B. (1997). A model for p53-induced apoptosis. *Nature*.

[5] Soengas M. S., Alarcón R. M., Yoshida H. (1999). Apaf-1 and caspase-9 in p53-dependent apoptosis and tumor inhibition. *Science*.

[6] Evan G. I., Wyllie A. H., Gilbert C. S. (1992). Induction of apoptosis in fibroblasts by c-myc protein. *Cell*.

[7] Furuya Y., Lundmo P., Short A. D., Gill D. L., Isaacs J. T. (1994). The role of calcium, pH, and cell proliferation in the programmed (apoptotic) death of androgen-independent prostatic cancer cells induced by thapsigargin. *Cancer Research*.

[8] Blattner W. A., McGuire D. B., Mulvihill J. J., Lampkin B. C., Hananian J., Fraumeni J. F. (1979). Genealogy of cancer in a family. *The Journal of the American Medical Association*.

[9] Srivastava S., Zou Z., Pirollo K., Blattner W., Chang E. H. (1990). Germ-line transmission of a mutated p53 gene in a cancer-prone family with Li-fraumeni syndrome. *Nature*.

[10] Sherif Z. A., Sultan A. S. (2013). Divergent control of cav-1 expression in non-cancerous Li-fraumeni syndrome and human cancer cell lines. *Cancer Biology and Therapy*.

[11] Kaminker P. G., Kim S.-H., Taylor R. D. (2001). TANK2, a new TRF1-associated poly(ADP-ribose) polymerase, causes rapid induction of cell death upon overexpression. *The Journal of Biological Chemistry*.

[12] Palo A. K., Sahu P., Choudhury R. C. (2005). Etoposide-induced cytogenotoxicity in mouse spermatogonia and its potential transmission. *Journal of Applied Toxicology*.

[13] Pines A., Mullenders L. H., van Attikum H., Luijsterburg M. S. (2013). Touching base with PARPs: moonlighting in the repair of UV lesions and double-strand breaks. *Trends in Biochemical Sciences*.

[14] Green D. R., Walczak H. (2013). Apoptosis therapy: driving cancers down the road to ruin. *Nature Medicine*.

[15] Pirollo K. F., Bouker K. B., Chang E. H. (2000). Does p53 status influence tumor response to anticancer therapies?. *Anti-Cancer Drugs*.

[16] Tang D., Lahti J. M., Grenet J., Kidd V. J. (1999). Cycloheximide-induced T-cell death is mediated by a Fas-associated death domain-dependent mechanism. *Journal of Biological Chemistry*.

[17] Sun X.-M., MacFarlane M., Zhuang J., Wolf B. B., Green D. R., Cohen G. M. (1999). Distinct caspase cascades are initiated in receptor-mediated and chemical-induced apoptosis. *Journal of Biological Chemistry*.

[18] Sherif Z. A., Danielsen M. (2006). Balanced t(11;15)(q23;q15) in a *TP*53^+/+^ breast cancer patient from a Li-Fraumeni syndrome family. *Cancer Genetics and Cytogenetics*.

[19] Miyashita T., Reed J. C. (1995). Tumor suppressor p53 is a direct transcriptional activator of the human *bax* gene. *Cell*.

[20] Yin C., Knudson C. M., Korsmeyer S. J., Van Dyke T. (1997). Bax suppresses tumorigenesis and stimulates apoptosis in vivo. *Nature*.

[21] Gil-Gómez G., Berns A., Brady H. J. M. (1998). A link between cell cycle and cell death: Bax and Bcl-2 modulate Cdk2 activation during thymocyte apoptosis. *The EMBO Journal*.

