# Supplementary material for: Caspase Activation and Aberrant Cell Growth in a p53+/+ Cell Line from a Li-Fraumeni Syndrome Family
Source: Genet Res Int. 2015 Mar 18;2015:789201. doi: 10.1155/2015/789201 (PMC4381654; doi:10.1155/2015/789201)
Supplement: Supplementary file 1 — This supplementary material contains raw data for the apoptotic assays induced by caspase (60-minute reaction in Table 1, and 120-minute reaction in Table 2) and Annexin V (Table 3). Table 4 provides data for the cell proliferation assay (Table 4). Standard errors and calculated probabilities were derived from these data sets. [file 789201.f1.docx]

Supplementary Supporting Raw Data

**Caspase Activity Assay**
Apoptosis was induced by adding 150 μM (final concentration) Etoposide into culture medium for various hours as indicated. Medium was replaced with fresh etoposide every 24 hours. Cells were collected by trypsinization. Apoptosis was analyzed using Caspase-3 Fluorometric Assay Kit from Biovision (after 60 minutes and 120 minutes after the reaction) according to the the manufacturer’s instructions. Results were analyzed by fluorometer. Data were shown as Relative Fluorescence Units in replicates of three.

**Table 1. Caspase Activity Assay
60 Minutes Reaction
WT-2852**

|  |  | **60 Mts** | **60 Mts** | **60 Mts** |  |  |  |
| --- | --- | --- | --- | --- | --- | --- | --- |
| Induction (Hrs) |  | WT-2852 | WT-2852 | WT-2852 | **Mean** | **STD** | **STE** |
|  | 0 | 1536 | 1505 | 1499 | 1513.333 | 19.85783 | 11.46492 |
|  | 24 | 8640 | 8444 | 8534 | 8539.333 | 98.10878 | 56.64313 |
|  | 48 | 4850 | 4760 | 4889 | 4833 | 66.1589 | 38.19686 |
|  | 72 | 3882 | 4000 | 3698 | 3860 | 152.1972 | 87.87112 |

**60 Minutes Reaction
MT-2852**

| **60 Mins** | **60 Mins** | **60 Mins** |  |  |  |
| --- | --- | --- | --- | --- | --- |
| MT-2673 | MT-2673 | MT-2673 | **Mean** | **STD** | **STE** |
| 1786 | 1788 | 1789 | 1787.667 | 1.414214 | 0.816497 |
| 3610 | 3678 | 3649 | 3645.667 | 48.08326 | 27.76088 |
| 3116 | 3255 | 3185 | 3185.333 | 98.28784 | 56.74651 |
| 2522 | 2399 | 2461 | 2460.667 | 86.97413 | 50.21454 |

**Table 2. Caspase Activity Assay
120 Minutes Reaction
WT-2852**

| **120 Mins** | **120 Mins** | **120 Mins** |  |  |  |
| --- | --- | --- | --- | --- | --- |
| WT-2852 | WT-2852 | WT-2852 | **Mean** | **STD** | **STE** |
| 1606 | 1606 | 1612 | 1608 | 3.464102 | 2 |
| 14028 | 14000 | 13567 | 13865 | 258.455 | 149.2191 |
| 8040 | 8000 | 8053 | 8031 | 27.62245 | 15.94783 |
| 6324 | 6783 | 6500 | 6535.667 | 231.5693 | 133.6966 |

**120 Minutes Reaction
MT-2673**

| **120 Mins** | **120 Mins** | **120 Mins** |  |  |  |
| --- | --- | --- | --- | --- | --- |
| MT-2673 | MT-2673 | MT-2673 | **Mean** | **STD** | **STE** |
| 2628 | 2601 | 2605 | 2611.333 | 14.57166 | 8.412953 |
| 5796 | 5801 | 5890 | 5829 | 52.88667 | 30.53413 |
| 4828 | 4699 | 4901 | 4809.333 | 102.2855 | 59.05459 |
| 3957 | 4002 | 4005 | 3988 | 26.88866 | 15.52417 |

**Annexin V Apoptosis Assay**Apoptosis was induced by adding 100 uM (final concentration) of etoposide into culture medium containing non-cancerous LFS fibroblasts 2852 and 2673, which were incubated for various hours as indicated. Apoptosis was analyzed using Annexin V-FITC Apoptosis Assay Kit (BioVision, Cat.# K101-100) according to the instructions in the kit. Results as shown in line graph **(3A)** and bar graph **(3B)** were analyzed on FACScan with 10000 cells being counted from each sample. A representative experiment of three independent replicates is shown.

**Table 3. Annexin V Apoptosis Assay
WT-2852 and MT-2673**

| Induction (hrs) | WT-2852 | WT-2852 | WT-2852 | MT-2673 | MT-2673 | MT-2673 |
| --- | --- | --- | --- | --- | --- | --- |
| 0 | 2.97 | 3.02 | 3.12 | 2.55 | 2.99 | 2.69 |
| 24 | 4.94 | 4.54 | 4.9 | 4.22 | 4.33 | 4.01 |
| 48 | 15.43 | 14.9 | 15.25 | 4.97 | 5.01 | 4.99 |
| 72 | 44.9 | 43.9 | 46.98 | 14.19 | 14.78 | 14.91 |

**Annexin V Apoptosis Assay
Standard Error Calculations**

|  | WT-2852 | WT2852 | WT-2852 | MT-2673 | MT-2673 | MT-2673 | 2852 | 2673 |
| --- | --- | --- | --- | --- | --- | --- | --- | --- |
|  | MEAN | STD | STE | MEAN | STD | STE | Expected | Expected |
| 0 | 3.03666 | 0.07637 | 0.044096 | 2.74333 | 0.224796 | 0.12978 | 17.0708 | 6.63666 |
| 24 | 4.79333 | 0.22030 | 0.127192 | 4.18666 | 0.162583 | 0.09386 | 17.0708 | 6.63666 |
| 48 | 15.1933 | 0.26950 | 0.155599 | 4.99 | 0.02 | 0.01154 | 17.0708 | 6.63666 |
| 72 | 45.26 | 1.57124 | 0.907157 | 14.6266 | 0.38371 | 0.22153 | 17.0708 | 6.63666 |

**Table 4. Cell Proliferation Assay
WT-2852 and MT-2673**

A =2852, B =2673

|  | **A1** | **A2** | **A3** | **A4** |  | **B1** | **B2** | **B3** | **B4** | **A  Cells** | **B  Cells** |
| --- | --- | --- | --- | --- | --- | --- | --- | --- | --- | --- | --- |
| DO | 0.236 | 0.239 | 0.237 | 0.239 |  | 0.269 | 0.264 | 0.271 | 0.265 | 0.238 | 0.267 |
| D1 | 0.269 | 0.268 | 0.27 | 0.266 |  | 0.291 | 0.303 | 0.302 | 0.298 | 0.268 | 0.299 |
| D2 | 0.363 | 0.405 | 0.363 | 0.344 |  | 0.376 | 0.464 | 0.362 | 0.378 | 0.369 | 0.395 |
| D3 | 0.895 | 0.762 | 0.899 | 0.752 |  | 0.597 | 0.565 | 0.581 | 0.572 | 0.827 | 0.579 |
| D4 | 1.439 | 1.549 | 1.552 | 1.453 |  | 0.699 | 0.733 | 0.706 | 0.699 | 1.498 | 0.709 |

^a^Numbers under A1-A4 represent the O.D. readings of 4 individual "A" cell wells. Numbers under B1-B4 represent the O.D. readings of 4 individual "B" cell wells. Numbers under A cells and B cells are the average of 4 readings for each cell type. D0-D4 indicates the days the measurements were performed.
